# Safety and immunogenicity of multi-antigen AMA1-based vaccines formulated with CoVaccine HT™ and Montanide ISA 51 in rhesus macaques

**DOI:** 10.1186/1475-2875-10-182

**Published:** 2011-07-04

**Authors:** Kwadwo A Kusi, Edmond J Remarque, Vanessa Riasat, Vanessa Walraven, Alan W Thomas, Bart W Faber, Clemens HM Kocken

**Affiliations:** 1 Department of Parasitology, Biomedical Primate Research Centre, Postbox 3306, 2280 GH, Rijswijk, The Netherlands; 2 Department of Immunology, Noguchi Memorial Institute for Medical Research, College of Health Sciences, University of Ghana, P.O. Box LG581, Legon, Accra, Ghana; 3 Department of Medical Microbiology, Haga Hospital, 2545 CH, The Hague, The Netherlands; 4 Vlietland Ziekenhuis, 3118 JH, Schiedam, The Netherlands

## Abstract

**Background:**

Increasing the breadth of the functional antibody response through immunization with *Plasmodium falciparum *apical membrane antigen 1 (*Pf*AMA1) multi-allele vaccine formulations has been demonstrated in several rodent and rabbit studies. This study assesses the safety and immunogenicity of three *Pf*AMA1 Diversity-Covering (DiCo) vaccine candidates formulated as an equimolar mixture (DiCo mix) in CoVaccine HT™ or Montanide ISA 51, as well as that of a *Pf*AMA1-MSP1_19 _fusion protein formulated in Montanide ISA 51.

**Methods:**

Vaccine safety in rhesus macaques was monitored by animal behaviour observation and assessment of organ and systemic functions through clinical chemistry and haematology measurements. The immunogenicity of vaccine formulations was assessed by enzyme-linked immunosorbent assays and *in vitro *parasite growth inhibition assays with three culture-adapted *P. falciparum *strains.

**Results:**

These data show that both adjuvants were well tolerated with only transient changes in a few of the chemical and haematological parameters measured. DiCo mix formulated in CoVaccine HT™ proved immunologically and functionally superior to the same candidate formulated in Montanide ISA 51. Immunological data from the fusion protein candidate was however difficult to interpret as four out of six immunized animals were non-responsive for unknown reasons.

**Conclusions:**

The study highlights the safety and immunological benefits of DiCo mix as a potential human vaccine against blood stage malaria, especially when formulated in CoVaccine HT™, and adds to the accumulating data on the specificity broadening effects of DiCo mix.

## Background

The development of an effective malaria vaccine remains an important public health objective for disease control in endemic areas. Vaccine strategies that control or prevent blood stage infection may be most desirable since blood stage parasites are responsible for clinical symptoms of the disease. Current knowledge of *Plasmodium falciparum*, the parasite responsible for the most severe form of disease suggests that a potentially effective vaccine would likely include multiple antigens, preferably expressed in different stages of the parasite's life cycle. Essential *P. falciparum *antigens that are currently being considered as subunit vaccine candidates include apical membrane antigen 1 (AMA1) and merozoite surface protein 1 (MSP1). AMA1 is highly polymorphic and is found in both merozoite and sporozoite stages of the parasite [1-4]. It is initially expressed as an 83 kDa precursor protein in the micronemes and undergoes an N-terminal prosequence cleavage to form the 66 kDa antigen at the same site [5]. AMA1 translocates to the parasite membrane surface at the time of red cell invasion, and plays a key role in the invasion process [5-9]. The AMA1 ectodomain, which is the vaccine target, is shed as 44 and 48 kDa alternate antigens before the parasite enters the red cell [5,10]. The ectodomain has 16 cysteine residues that form disulphide bonds to divide the antigen's tertiary structure into three different but interactive domains [11].

MSP1, another important vaccine candidate, is the parasite major surface antigen that also plays a role in the red cell invasion process and is dimorphic [12-15]. MSP1 is expressed as a precursor protein of approximately 200 kDa on the surface of developing merozoites [16]. It is proteolytically processed into several fragments at the time of schizont rupture and red cell invasion. The 42 kDa fragment, which is a vaccine candidate, is subsequently processed into 33 kDa and 19 kDa fragments [17,18]. The 19 kDa fragment (MSP1_19_), which is a major vaccine target, remains anchored to the merozoite surface and can be detected in early red cell stages of the parasite [19,20]. All other MSP1 fragments are shed as a peptide complex prior to red cell invasion.

These antigens have demonstrable vaccine properties in rodent and non-human primate models as well as in *in vitro *systems [21-27]. Their vaccine potential, which is exhibited mainly through antibody-mediated mechanisms [28,29], is however limited by allelic polymorphism [24,26,30-32]. Multi-allele vaccination studies, mostly in rabbits and rodents, have however shown promise in overcoming the strain-specific effects of polymorphism on immune responses to these antigens. This strategy informed the design, expression and purification of three Diversity-Covering (DiCo) *P. falciparum *AMA1 (*Pf*AMA1) antigens based on the sequences of 355 naturally occurring *Pf*AMA1 alleles [33]. The DiCo vaccine candidate is an equimolar mixture (DiCo mix) of the three DiCo antigens, hence apart from the design strategy which is to cover polymorphism, mixing of the three DiCo antigens cover polymorphism on a second level. DiCo mix formulated with either Montanide ISA 51 or CoVaccine HT™ as adjuvant has been shown to induce rabbit humoral responses with similar high inhibitory capacities against multiple parasite strains *in vitro *[33,34]. Another strategy for dampening the effects of polymorphism on *Pf*AMA1 responses is to combine *Pf*AMA1 candidates with other highly immunogenic candidates that show limited polymorphism. This second strategy may involve mixing of the separately expressed and purified vaccine component antigens, or the expression and purification of component antigens as a single chimeric protein. In one such study, rabbits were immunized with *Pf*MSP1_19 _(Welcome strain) and *Pf*AMA1 (domains I and II of the FVO strain) proteins formulated as a mixture or the expressed chimeric proteins in Montanide ISA720 and functionality of induced IgGs was tested against three parasite strains (FCR3, HB3, 3D7). IgG responses to *Pf*AMA1 alone or the *Pf*AMA1/*Pf*MSP1_19 _vaccine products showed signs of strain specificity in functional assays, while responses to products containing *Pf*MSP1_19 _alone showed limited strain-specificity [35]. Challenge studies in rodents immunized with a cocktail of *Plasmodium chabaudi *AMA1 and MSP1_42 _antigens also showed a greater reduction in peak parasitaemia compared to separate immunizations with the single antigens [36]. Another study involving a combination vaccine that consists of *Pf*AMA1 and *Pf*MSP1_42 _yielded responses that were directed against both antigens, though protection induced by the combination vaccine was not superior to that induced by the *Pf*AMA1 vaccine alone [27].

Most pre-clinical evaluations of these immunization strategies are done in rodents and rabbits. Vaccine evaluation in a monkey model, though not legally mandatory, may be an important step as it could yield additional data on vaccine safety and immunogenicity [37]. The *in vitro *parasite growth inhibition assay may have some limitations when used for assessment and down-selection of potentially important candidates, whose vaccine effects might be mediated by any of a number of different mechanisms. It nevertheless gives some idea of *in vivo *functionality, especially for antibody responses that are known to have a direct blocking effect on antigen processing and/or host cell invasion. In the current study the capacity of the DiCo mix and *Pf*AMA1-MSP1_19 _candidates, formulated in either Montanide ISA 51 or CoVaccine HT™ as adjuvants, to induce functional broad-strain antibody responses in non-human primates was examined. The safety and tolerability of these adjuvant formulations were also assessed as a proxy to effects that are likely to be observed in clinical testing of these candidates.

## Methods

### Antigens

All antigens were expressed as recombinant proteins in *Pichia pastoris *systems and details of gene expression and protein production have already been described elsewhere [33,35,38]. Natural *Pf*AMA1 alleles comprise the full length ectodomain (25-545) of the CAMP [GenBank:M58545], 3D7 [GenBank:U65407), HB3 [GenBank:U33277] and FVO [GenBank:AJ277646] strains of *P. falciparum*. DiCo proteins consist of amino acids 97 - 545 of the AMA1 ectodomain (domains I, II and III, without the signal sequence). The *Pf*AMA1-MSP1_19 _fusion protein (designated AM) consists of amino acids 106 - 442 (domains I and II) of the FVO AMA1 ectodomain fused with a mutant form of *Pf*MSP1_19 _(amino acids 1526 - 1621) of the Wellcome strain of *P. falciparum *[35]. All antigens were recognized by the reduction-sensitive rat monoclonal 4G2 antibody suggesting a correct folding of proteins. All antigens were devoid of N-glycosylation sites and the molecular size of the fusion protein product is comparable to that of any of the AMA1 proteins.

### Animal welfare and ethical clearance

Animals used in this study were captive-bred for research purposes. Experimentation and housing were at the Biomedical Primate Research Centre (BPRC) animal facility in Rijswijk, the Netherlands, in accordance with Dutch laws and European Acts (directive 86/609/EEC) on animal experimentation. The BPRC is compliant with recommendations of the Weatherall report on the use of non-human primates in research [39]. The study was approved by an independent ethics committee at BPRC, constituted in accordance with Dutch law on animal experimentation. To minimize discomfort to animals, immunization and blood sampling were all done under ketamine sedation. The study involved three experimental groups, each with six rhesus monkeys. Animals were assigned in a manner that ensured that age, weight and sex were similar amongst groups, and treatments were randomly assigned to groups.

### Vaccine formulation, immunization and bleeding

Two of the three groups of rhesus macaques were immunized with DiCo mix in either Montanide ISA 51 (Seppic, Paris, France) or CoVaccine HT™ (Protherics Medicines Development Limited, A BTG International Group Company, London, UK) as adjuvant, and the third group was immunized with the AM fusion protein formulated in Montanide ISA 51. Formulations were made under sterile conditions according to the respective adjuvant manufacturers' protocols. For Montanide ISA 51 formulations, 276 μl of antigen solution (130 μg/ml of DiCo mix or 217 μg/ml of AM) was added to 324 μl of adjuvant and the mixture emulsified by 20 passages through a Teflon-coated 22 gauge syringe-coupling piece. Five hundred microlitres (500 μl) of this formulation was administered per animal. For CoVaccine HT™ formulation, 300 μl of DiCo mix (120 μg/ml in saline) was mixed with an equal volume of adjuvant to a sucrose fatty acid sulphate esters (SFASEs) concentration of 20 mg/ml. Five hundred microlitres (500 μl) of the resulting mixture with 10 mg SFASEs was administered after gentle mixing. Animals were immunized intramuscularly in alternating legs on days 0 (left), 28 (right) and 56 (left) with either 30 μg of DiCo mix (two groups, designated DiCo/ISA and DiCo/HT) or 50 μg AM fusion protein (1 group, designated AM/ISA). Small aliquots of blood (between 2 ml and 7 ml) were taken on days 0, 1, 7, 14, 28, 29, 35, 42, 56, 57, 63 and 70 for clinical chemistry, serology and haematology, and larger volumes (up to 27 ml) were taken on Day 70 for serology, IgG isolation and subsequent *in vitro *testing.

### Safety monitoring

All treatment groups were monitored for safety by the assessment of local reactions (Draize scores), behaviour, appetite, stool and bodyweight as well as by clinical chemistry and haematology. Injection sites were inspected for local reactions on days 0, 1, 7 and 14 after each vaccination. Animals were also monitored on a daily basis by the caretakers and injection sites were frequently inspected. Clinical chemistry to assess organ and systemic functions was performed with a Cobas Integra 400 analyzer (Roche Diagnostics, Basel, Switzerland) according to standard methods and compared with normal values based on cumulative data from healthy animals within the same animal facility. Parameters measured include alkaline phosphatase, alanine transaminase, aspartate transaminase, bilirubin, lactate dehydrogenase, gamma glutamyl transpeptidase, cholesterol, glucose, iron, potassium, sodium, calcium, phosphate, chloride, bicarbonate, albumin, creatinine, total protein and urea. Haematology was performed with an automated analyser (Sysmex XT 2000iV platform; Goffin Meyvis, Etten-Leur, the Netherlands) and measurements were made for the red blood cell fraction (haemoglobin levels, erythrocyte count, haematocrit, mean corpuscular volume, mean corpuscular haemoglobin) and for the white blood cell fraction (white blood cell, lymphocyte, neutrophil, monocyte, eosinophil and basophil counts) as well as for platelets and mean platelet volume.

Safety assessments were made before immunization and one day, one week and two weeks following each immunization. Since all parameters had returned to normal values by day 70, body weight was the only parameter monitored on days 99 and 126.

### Immunological assessment of vaccine responses

Anti-AMA1 IgG levels in serum samples from blood drawn before immunization on days 0, 28 and 56, as well as samples taken on days 14, 42, 70, 99 and 126 were determined using an indirect ELISA. IgGs were measured against seven different *Pf*AMA1 alleles (the three DiCo antigens and recombinant *Pf*AMA1 alleles from the FVO, HB3, 3D7 and CAMP parasite strains). Anti-MSP1_19 _IgG levels in the AM vaccine group were measured with MSP1_19 _as capture antigen. Briefly, plates were coated with 0.5 μg/ml of the relevant antigen in PBS and subsequently blocked with 3% BSA in PBS with 0.05% Tween 20. Serum samples were diluted in PBS containing 0.1% BSA and 0.05% Tween-20. A pool of hyperimmune sera with high AMA1 and MSP1_19_-specific antibody titres was included on each plate as a standard calibrator. Antibodies were detected with an affinity-purified anti-human IgG coupled to alkaline phosphatase. Plates were developed with 1 mg/ml para-nitrophenyl phosphate, and the absorbance at 405 nm was measured. Antibody titres were subsequently expressed in arbitrary units (AU), with 1AU being equivalent to the reciprocal dilution at which an absorbance of 1 over background is achieved.

Antibody functionality was assessed by *in vitro *growth inhibition assays using the FCR3 (with one prodomain amino acid difference from AMA1 of the FVO strain [GenBank:M34553]), NF54 (parent clone of the 3D7 clone, [GenBank:U65407]) and HB3 [GenBank:U33277] culture-adapted parasite strains as has been previously described [33,38]. Antibodies were purified from day 70 sera using Protein A Sepharose (GE Healthcare, Etten-Leur, The Netherlands) columns and used at a final concentration of 10 mg/ml. Parasite strains were verified by restriction fragment length analysis and cultures were shown to be negative for mycoplasma.

### Data analyses

Clinical chemistry and haematological data assessment was based on normal reference values calculated from cumulative data of similar measurements in healthy animals within the same facility. IgG titres determined by ELISA for the different immunization groups were log-transformed to achieve normality and compared by one-way analysis of variance (ANOVA). Tukey HSD post hoc test with correction for multiple comparisons was used for pair-wise comparison of IgG titres in the same immunization groups against different capture antigens. Student *t *tests were used for the pair-wise comparison of GIA data between immunization groups against the same parasite strain, while the Tukey HSD post hoc test was used to compare data for the same immunization group against different strains. ELISA antibody titres and GIA data from day 70 sera (or purified IgG) are also presented as dotplots superimposed with boxplots showing the median inhibition as well as the first and third quartiles per treatment group. Plot symbols represent individual animals within the same treatment group. All graphics and analyses were performed using the R statistical package (R Development Core Team, 2010, version 2.12.1).

## Results

### Safety monitoring

Vaccine formulations with both adjuvants were generally well tolerated. All animals maintained body weights within reference ranges of normal values for the entire duration of the study. There were also no major changes in behaviour, appetite or stool over the period of observation. Apart from palpable inguinal lymph nodes there were no notable local reactions (oedema, erythema, indurations) in all 18 study animals. Levels of aspartate transaminase in the DiCo/ISA immunization group as well as alanine transaminase and bilirubin in the AM/ISA group showed slight increases on the days following immunization but returned to normal levels within a week. There was an increase in creatinine levels a day after immunization in the DiCo/HT group but these also returned to normal levels within a week. Creatinine levels in the DiCo/ISA group were however above normal values at the start of the study and remained at similar high levels throughout the observation period. Levels of blood iron decreased on the days following immunization in the DiCo/HT group but also returned to normal levels within a week. All other measured clinical chemistry parameters were within normal reference ranges throughout the 70-day observation period.

Of the parameters measured for haematology, increases in neutrophil count (and hence white blood cell count) were observed a day after each immunization, but these again returned to normal values within a week. All other measured parameters were between normal ranges throughout the study.

Generally, local reactions were limited to mild reddening of the injection site area, and these resolved within a few days. One animal in the AM/ISA group however developed stiffness in the upper leg muscle to a degree that limited movement of the left leg. This adverse event was first observed 14 days after the last immunization (day 70) and had not resolved on day 126. Given this observation, which lasted for more than 56 days, the amount of discomfort to this animal was rated moderate to serious.

### Elisa antibody responses

**Figure 1:**
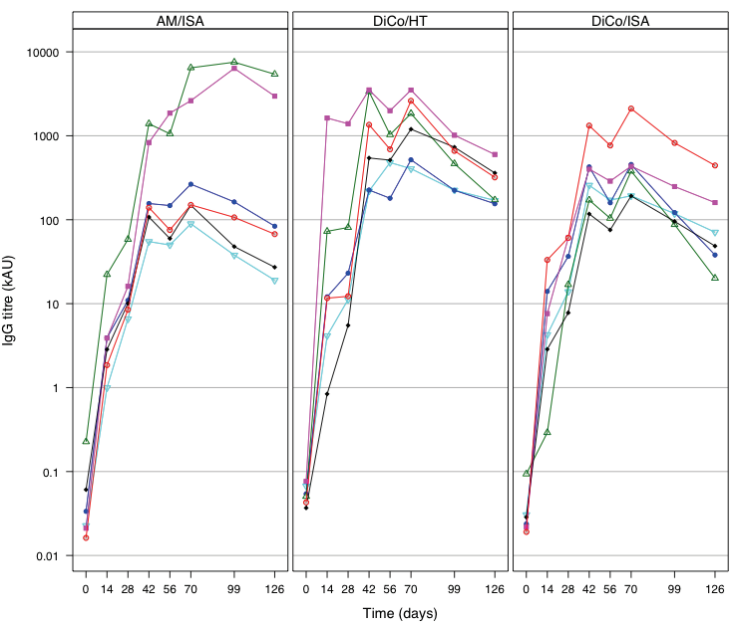
**Anti-FVO AMA1 IgG titres in sera of animals immunized with AMA1-based vaccines**. Three groups of six rhesus monkeys each were immunized with *Pf*AMA1-based formulations on days 0, 28 and 56. One group was immunized with DiCo mix formulated in CoVaccine HT™ (DiCo/HT), the second group with DiCo mix in Montanide ISA 51 (DiCo/ISA), and the third group with an AMA1-MSP1_19 _fusion protein in Montanide ISA 51 (AM/ISA). IgG titres in samples taken prior to each immunization, as well as in samples drawn on days 14, 42, 70, 99 and 126, were measured by an indirect ELISA. Similar patterns were observed for IgG measurements against six other *Pf*AMA1 alleles (vaccine antigens DiCo 1, DiCo 2 and DiCo 3 as well as *Pf*AMA1 from the HB3, 3D7 and CAMP parasite strains). For each panel, the different colours/symbols represent individual animals.

**Figure 2:**
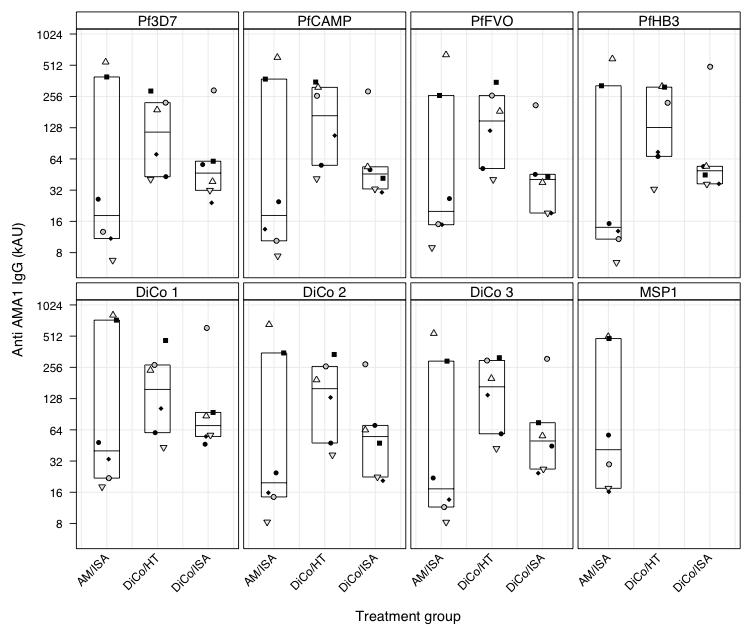
**IgG titres in day 70 sera from all three immunization groups**. Anti-*Pf*AMA1 titres in all immunization groups were measured against seven allelic vaccine antigens (DiCo 1, DiCo 2 and DiCo 3 as well as *Pf*AMA1 from the FVO, HB3, 3D7 and CAMP parasite strains) while anti- MSP1_19 _IgG levels in the AM/ISA group were measured against *Pf*MSP1_19_. For each immunization group, plot symbols represent data from individual animals in all panels. Plot symbols are also the same as those used for the same animals in Figure 1.

IgG levels against seven AMA1 alleles and MSP1_19 _at all sampling time points were determined using a harmonized ELISA. The three vaccine formulations induced appreciable levels of antibodies against the tested antigens and titres against all antigens increased in a similar manner and generally peaked on day 70, two weeks after the final vaccine injections were given. IgG titres against the FVO AMA1 allele at all time-points are presented in Figure 1 and day 70 titres against all antigens are presented in Figure 2. Beyond day 70, IgG titres in the two DiCo mix immunization groups showed a decline that was statistically significantly lower on day 126 compared to day 70 levels (p < 0.05 for all AMA1 antigens, Student *t *test). Both the anti-AMA1 and anti-MSP1_19 _levels for the AM/ISA group were however not significantly different on days 70 and 126 (p > 0.05, Student *t *test).

Vaccine-induced IgG titres against all seven AMA1 capture antigens were generally highest in the DiCo/HT group by day 70. A comparison of the geometric mean titres, either for the same immunization group against all AMA1 antigens, or for all groups against the same capture antigen, showed that there were no statistically significant differences (P > 0.05, one-way ANOVA). Anti-AMA1 IgG titres varied most amongst animals in the AM group, with four of the six animals having very low IgG titres against all antigens and the other two having exceptionally high titres (Figure 2).

Anti-MSP1_19 _IgG titres were determined only for the AM group (Figure 2), and these were statistically comparable to anti-AMA1 IgG titres against all seven AMA1 capture antigens for the same group (p = 0.99, one-way ANOVA). The four animals with low anti-AMA1 IgG levels in this group also had the lowest anti-MSP1_19 _IgG levels.

### *In vitro *growth inhibition data

**Figure 3:**
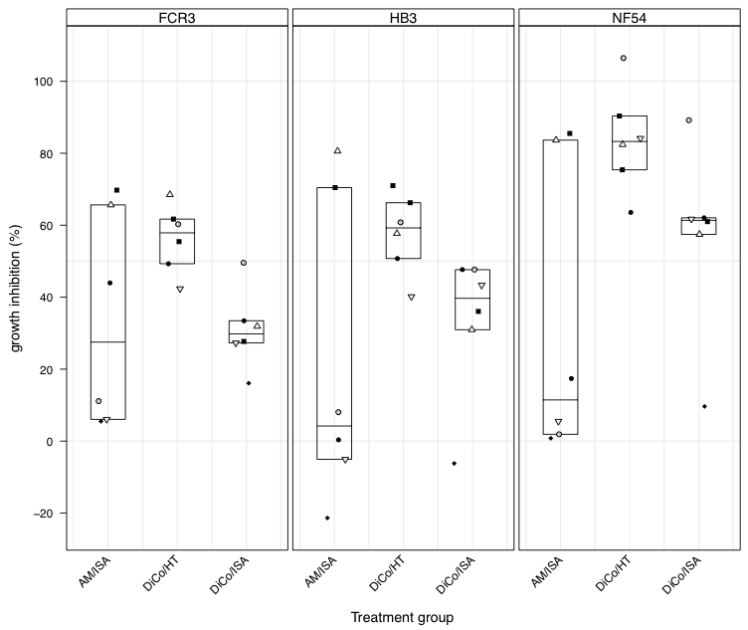
***In vitro *parasite growth inhibition levels of protein A-purified IgGs from day 70 samples**. IgGs were tested in triplicate at a final concentration of 10 mg/ml against the FCR3, HB3 and NF54 culture-adapted strains of *P. falciparum*. The data presented is the average of two independent assays, and plot symbols represent data from individual animals within the same immunization group in all panels. Plot symbols are also the same as that used for the same animals in other figures.

**Figure 4:**
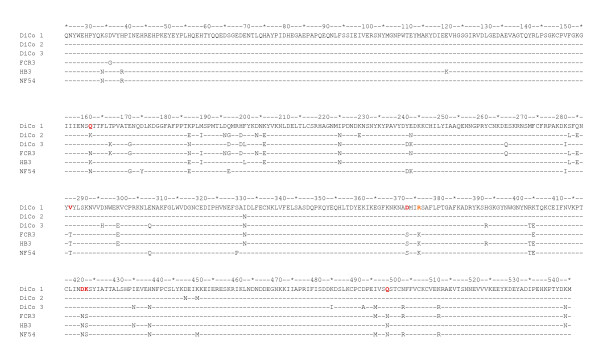
**Protein sequence (aa 25 - 545) alignments for DiCo antigens and parasite AMA1 alleles**. AMA1 protein sequences of malaria parasites used for *in vitro *growth inhibition assays were accessed from the GenBank database. The DiCo sequences contain point mutations at the cleavage (K376R, indicated in orange) and potential N-glycosylation (N162Q, T288V, S373D, N422D, S423K, N499Q, indicated in red) sites and differ from parasite AMA1 sequences at these sites. Amino acid residues 25 to 96 represent the prodomain of the AMA1 transmembrane protein, residues 97 - 303 represent domain I, residues 304 - 440 represent domain II and residues 441 - 545 represent domain III of the protein ectodomain.

*In vitro *growth inhibition assays were performed on the FCR3, HB3 and NF54 culture-adapted strains of *P. falciparum *using protein G-purified IgGs from day 70 sera. Purified IgGs were tested against each parasite strain at a final concentration of 10 mg/ml, and the data, presented in Figure 3, is the average of two independent assays per parasite strain. The amino acid sequence differences between the three DiCo antigens and the AMA1 alleles expressed by the *P. falciparum *strains used in assays is presented in Figure 4. All animals in the DiCo/HT group responded well to the vaccine by eliciting levels of IgG that substantially inhibited parasites, while IgGs elicited by one animal in the DiCo/ISA group and four animals in the AM/ISA group had growth inhibition levels lower than 20% against all three strains. Growth inhibition levels for the AM/ISA group therefore had a very wide range.

At 10 mg/ml, IgGs from the DiCo/HT group had mean growth inhibition (56.3%, 57.8% and 83.7% for FVO, HB3 and NF54 strains, respectively) that was significantly higher than that of IgGs from the DiCo/ISA group (31.0%, 33.2% and 56.8% for FVO, HB3 and NF54 strains, respectively) against all three parasite strains (p = 0.002 for FCR3 strain, p = 0. 033 for HB3 and p = 0.006 for NF54, student *t *test). Mean growth inhibition was significantly higher for the DiCo/HT group compared to the AM/ISA group (32.5%) against the NF54 strain alone (p = 0.026), while differences between the DiCo/ISA and AM/ISA groups against all three strains were not statistically significant (p > 0.05 in all cases). It must be noted that IgGs from one animal in the DiCo/HT group completely inhibited the NF54 strain, with the average of the two measurements being above 100%, an obvious artefact.

Comparison of mean growth inhibition per immunization group amongst the three parasite strains showed that IgGs from the DICo/HT group inhibited NF54 parasites better than the other two strains (p = 0.003 against FCR3 and p = 0.005 against HB3, Tukey HSD). Mean inhibition for this group however did not differ between HB3 and FCR3 parasites (p = 0.973, Tukey HSD). Mean inhibition against the three parasite strains was similar for both the DiCo/ISA and AM/ISA groups (p = 0.85 for AM/ISA and p = 0.076 for DiCo/ISA, one-way ANOVA). In this study, inhibition of the growth of the NF54 parasite strain was unexpectedly high in comparison with the other two strains.

## Discussion

Multi-allele and multi-antigen malaria vaccine approaches have shown potential in inducing antibody responses with broad-strain inhibitory capacity in a number of rodent and rabbit studies [33,34,40]. In the current study, the immunological benefits of such immunization strategies, as well as the safety of these formulations, were further analysed in a non-human primate model. Two different adjuvants were used for vaccine formulation in this study; Montanide ISA 51 (w/o) is a proprietary adjuvant from Seppic (Paris, France) and has been used in a number of human studies [41-43]. CoVaccine HT™ (o/w) is also a proprietary adjuvant developed by Protherics Medicines Development Limited, a BTG International Group company, and has recently entered human trials [44]. In the current study both adjuvant formulations were well tolerated as most of the parameters (chemistry and haematology) measured to assess organ and systemic functions were well within normal values for healthy animals within the same facility. Palpable inguinal lymph nodes were the only local reaction to vaccination observed in study animals. Mean levels of alanine transaminase in the AM/ISA immunization group and aspartate transaminase in the DiCo/ISA group increased a day after each immunization but were back to baseline within a week. Transient increases were seen in individual animals in all immunization groups but only those in groups immunized with Montanide ISA 51 showed mean levels higher beyond normal values. Hypotension and hypoxaemia induced following ketamine sedation have been associated with a release of these enzymes from the liver and heart muscle [45,46] and it is possible that Montanide ISA 51 enhanced this effect.

Mean levels of creatinine for the DiCo/ISA group were high throughout the study period, with four of the six animals in the group showing levels above normal prior to the first immunization and throughout the study. As these levels remained largely unchanged throughout the study, it suggests that treatments did not alter renal function. Increased neutrophil counts in the DiCo/HT group following immunization were concomitant with decreased levels of serum iron in the same group. This observation has also been made in previous studies and is suggestive of the possibility that these events are coupled. The limited leg movement experienced by one animal in the AM/ISA group was most likely due to physical injury from needles used to deliver the vaccine or for blood sampling and not vaccine-related. Moreover, such an outcome has not been observed in earlier studies in this lab with this adjuvant.

Previous studies with CoVaccine HT™ formulations at an SFASEs dose of 2 mg or 10 mg in rhesus monkeys [47,48] and at 2 mg or 4 mg SFASEs in rabbits [47] showed no local or systemic adverse events, while only minimal transient adverse events were seen in a small percentage of ferrets with formulations containing 0.125 - 4 mg of SFASEs ([47,49]). For Montanide ISA 51, though little to no reactogenicity has been reported in animal studies and in some human studies [41,50-52], other human studies have concluded that this adjuvant, with its current composition, might not be suitable for use in humans due to high reactogenicity [42,43].

Mean antibody responses against the AM/ISA vaccine were statistically similar on day 70 and 126 while responses against the two DiCo mix groups had declined significantly by day 126. This was due mainly to the exceptionally high titres in two of the six animals in the AM/ISA group (Figure 1) and does not reflect a more durable IgG response against the AM fusion protein compared to DiCo mix, especially since the four low responders in the AM group also showed titre decreases on day 126. The AM/ISA vaccine also induced appreciable levels of anti-MSP1_19 _IgG, especially in the two animals that also had the highest anti-AMA1 IgGs in this group. Vaccine design studies with MSP1_19 _fusion proteins have shown that IgG responses induced against the MSP1_19 _component increase several fold compared to IgG responses to formulations with MSP1_19 _alone [35,53], suggesting that fusion enhances antibody formation against the MSP1_19 _component.

Observations in ELISA were in agreement with the *in vitro *growth inhibition data as animals with high IgG levels had correspondingly high GIA activities and vice versa. Purified IgGs from all animals in the DiCo/HT group showed high mean growth inhibition of the three parasite strains compared to inhibition by IgGs in animals from the DiCo/ISA group (Figure 3). This suggests that the CoVaccine HT™ formulation was functionally superior and that this adjuvant may be most suitable for inducing the required high titres of functional IgGs. Inhibition of all three strains was similar for all IgGs from the DiCo/ISA or AM/ISA immunization groups (Figure 3). Inhibition of NF54 parasites by IgGs from the DiCo/HT group was however slightly higher compared to that of the two other strains (Figure 3). This can however not be attributed to greater sequence similarity between NF54 AMA1 and the DiCo antigens. Indeed the greatest sequence similarity for the AMA1 allele sequences indicated in Figure 4 is between the FVO AMA1 allele and DiCo 2 (12 amino acid differences apart from the one cleavage and six N-glycosylation site differences). This notwithstanding, the data demonstrate the specificity broadening benefits of DiCo mix formulations in non-human primates, and adds to the accumulating data on the strain-transcending properties of the DiCo strategy that have been demonstrated in rabbit studies [33,34].

Four animals in the AM/ISA group had very low IgG titres (Figure 2) with corresponding low inhibitory activities below 20%, especially against the HB3 and NF54 strains (Figure 3). The other two animals in this group had high vaccine responses in both ELISA and GIA (Figures 1 and 2) hence the weak responses in the four animals cannot be due to poor immunogenicity of the candidate antigens. Thus these animals were most likely non-responsive to immunization, and this makes it difficult to draw firm conclusions on the effectiveness of the AM fusion protein candidate in this study. Parasite inhibition studies done *in vitro *with antibodies raised in rabbits as well as challenge studies in mice have however shown that vaccines composed of AMA1 and MSP1 either as a mixture or a fusion protein product induced functional antibodies [35,36,54].

The data presented shows that both adjuvant formulations were well tolerated upon administration to rhesus macaques. The data further demonstrates the specificity broadening benefits of multi-allele formulations compared to single *Pf*AMA1 formulation in non-human primates [55,56], and adds to the accumulating data on the strain-transcending properties of the DiCo strategy. The three antigens, especially when formulated in CoVaccine HT™, induced IgG levels that inhibited multiple parasite strains *in vitro*. It is however difficult to draw firm conclusions on data from the AM fusion protein since most of the test animals immunized with this vaccine showed low vaccine responses. The DiCo antigens thus represent a unique strain-transcending strategy for developing a malaria blood stage vaccine with benefits for susceptible individuals in areas where *P. falciparum *is endemic.

## Competing interests

Four of the authors are in the process of obtaining a patent for the three synthetic Diversity-Covering (DiCo) AMA1 proteins. This does not alter their adherence to any Malaria Journal policies on sharing data and materials. All other authors have no competing Interests.

## Authors' contributions

Conceived and designed the experiments: EJR BWF CHMK AWT. Performed the experiments: KAK, VW. Analysed the data: KAK EJR BWF. Designed and produced recombinant proteins: BWF, VR. Wrote the paper: KAK EJR BWF CHMK AWT. All authors have read and approved the final manuscript.
